# Integrated genomic sequencing in myeloid blast crisis chronic myeloid leukemia (MBC-CML), identified potentially important findings in the context of leukemogenesis model

**DOI:** 10.1038/s41598-022-17232-w

**Published:** 2022-07-27

**Authors:** Golnaz Ensieh Kazemi-Sefat, Mohammad Keramatipour, Mohammad Vaezi, Seyed Mohsen Razavi, Kaveh Kavousi, Amin Talebi, Shahrbano Rostami, Marjan Yaghmaie, Bahram Chahardouli, Saeed Talebi, Kazem Mousavizadeh‬

**Affiliations:** 1 Department of Molecular Medicine, Faculty of Advanced Technologies in Medicine, Iran University of Medical Sciences, Tehran, Iran; 2 Cellular and Molecular Research Center, Iran University of Medical Sciences, Tehran, Iran; 3 Department of Medical Genetics, School of Medicine, Tehran University of Medical Sciences, Tehran, Iran; 4 Hematology, Oncology and Stem Cell Transplantation Research Center, Shariati Hospital, Tehran University of Medical Sciences, Tehran, Iran; 5 Oncopathology Research Center, School of Medicine, Iran University of Medical Sciences, Tehran, Iran; 6 Laboratory of Complex Biological Systems and Bioinformatics (CBB), Department of Bioinformatics, Institute of Biochemistry and Biophysics (IBB), University of Tehran, Tehran, Iran; 7 Department of Medical Genetics, Faculty of Medicine, Mashhad University of Medical Sciences, Mashhad, Iran; 8 Department of Medical Genetics, School of Medicine, Iran University of Medical Sciences, Tehran, Iran; 9 Department of Pharmacology, School of Medicine, Iran University of Medical Sciences, Tehran, Iran

**Keywords:** Cancer, Molecular medicine

## Abstract

Chronic myeloid leukemia (CML) is a model of leukemogenesis in which the exact molecular mechanisms underlying blast crisis still remained unexplored. The current study identified multiple common and rare important findings in myeloid blast crisis CML (MBC-CML) using integrated genomic sequencing, covering all classes of genes implicated in the leukemogenesis model. Integrated genomic sequencing via Whole Exome Sequencing (WES), Chromosome-seq and RNA-sequencing were conducted on the peripheral blood samples of three CML patients in the myeloid blast crisis. An in-house filtering pipeline was applied to assess important variants in cancer-related genes. Standard variant interpretation guidelines were used for the interpretation of potentially important findings (PIFs) and potentially actionable findings (PAFs). Single nucleotide variation (SNV) and small InDel analysis by WES detected sixteen PIFs affecting all five known classes of leukemogenic genes in myeloid malignancies including signaling pathway components (*ABL1*, *PIK3CB*, *PTPN11*), transcription factors (*GATA2*, *PHF6*, *IKZF1*, *WT1*), epigenetic regulators (*ASXL1*), tumor suppressor and DNA repair genes (*BRCA2*, *ATM*, *CHEK2*) and components of spliceosome (*PRPF8*). These variants affect genes involved in leukemia stem cell proliferation, self-renewal, and differentiation. Both patients No.1 and No.2 had actionable known missense variants on *ABL1* (p.Y272H, p.F359V) and frameshift variants on *ASXL1* (p.A627Gfs*8, p.G646Wfs*12). The *GATA2*-L359S in patient No.1, *PTPN11*-G503V and *IKZF1*-R208Q variants in the patient No.3 were also PAFs. RNA-sequencing was used to confirm all of the identified variants. In the patient No. 3, chromosome sequencing revealed multiple pathogenic deletions in the short and long arms of chromosome 7, affecting at least three critical leukemogenic genes (*IKZF1, EZH2*, and *CUX1*). The large deletion discovered on the short arm of chromosome 17 in patient No. 2 resulted in the deletion of *TP53* gene as well. Integrated genomic sequencing combined with RNA-sequencing can successfully discover and confirm a wide range of variants, from SNVs to CNVs. This strategy may be an effective method for identifying actionable findings and understanding the pathophysiological mechanisms underlying MBC-CML, as well as providing further insights into the genetic basis of MBC-CML and its management in the future.

## Introduction

Chronic Myeloid Leukemia (CML) is a unique model for the evolution of cancer and is classified as a triphasic myeloproliferative neoplasm based on clinical and pathological characteristics. Premalignant leukemia stem cells (LSCs) are, in fact, generated in the bone marrow nich by unknown mutagenesis processes^1,2^. In this step, the disease may be undetectable for a decade or more. Further oncogenic processes cause LSCs to develop into leukemia progenitor cells (LPCs)^3^. Leukemogenic alterations mainly affect five classes of regulatory proteins: signaling pathway components, transcription factors (TFs), epigenetic regulators (ERs), tumor suppressor genes (TSGs), and components of the spliceosome^4^.

Without therapeutic interventions or in the case of drug resistance, CML will progress to accelerated phase (AP) and then into an acute leukemia phase that is called blastic phase or blast crisis^5^. Even in the advent of Tyrosin Kinase Inhibitor therapy, blastic phase survival is less than 12 months. In general, blast crisis in chronic myeloid leukemia is still a fatal disease^6,7^.

Over the past decade using Massively Parallel Sequencing (MPS) or Next Generation Sequencing (NGS) the multi-dimensional genomic view with higher resolution of molecular profiling has become possible^8^. It was shown that the combination of exome, genome and RNA Sequencing data^9^, called integrative sequencing^10–12^ paves the way to better understand the mechanisms and pathogenesis of the cancer and can potentially improve the clinical management and finding new therapeutic targets specially in the advanced phase patients^13^.

In the present study, we conducted integrated genomic sequencing through an in-house filtering algorithm to uncover the leukemogenic important/actionable findings and investigate the myeloid leukemogenesis model in three Iranian myeloid blast crisis CML patients.

## Methods

### Subjects

Three patients (from 3 unrelated families) with the clinical diagnosis of blastic phase CML were recruited from the Shariati Hospital in Tehran, Iran. Inclusion criteria were based on clinical presentations and hematological findings in the peripheral blood and bone marrow, according to the WHO 2016-guideline^14^.

The study was approved by the Ethical Committee of the Iran University of Medical Sciences (Code: IR.IUMS.REC 1395.95-04-87-30235). All subjects and/or their legal guardian(s) gave their informed consent to participate. All methods, including obtaining informed consent, were carried out in compliance with the above-mentioned ethical standards and guidelines.

Whole Exome Sequencing (WES) along with Chromosome-seq and RNA Sequencing were performed on extracted DNA and RNA from peripheral blood samples in the blast crisis phase.

### Whole exome sequencing

Peripheral blood samples of patients were collected in EDTA containing tubes and used to extract genomic DNA by Blood SV-mini kit (GeneAll Biotechnology Co., LTD, South Korea) according to the manufacturer instruction. The extracted genomic DNA samples were subjected to WES using Agilent SureSelect V6-post Capture/Target Enrichment Kit. For the third patient TWIST Human Core Exome Kit (Twist Bioscience, USA) was used according to the manufacturer’s instruction. The enriched libraries were sequenced on Illumina platforms (Illumina Inc., CA, USA). Table S1 shows a summary report of data analysis metrics from the patients’ WES.

### WES data analysis (variant filtering)

Paired-end sequence reads were mapped to the refseq (NCBI) human reference genome (GRCh38 assembly) by BWA-MEM. Picard software was used to mark duplicate reads. Genome Analysis Tool Kit (GATK) software Mutect2 package was used to call single nucleotide variants (SNVs) and short insertions or deletions (Indels). The intronic variants were removed, however the 100 bp flanking each exon was included in VCF files using interval/bed file.

Several tools were used to annotate and interpret variants including CGI, Varsome, InterVar, CancerVar, and Franklin. The annotated VCF files were filtered to find Tier1 or Tier2 variants according to AMP classification and pathogenic (P), likely pathogenic (LP), or Leaning pathogenic variants based on ACMG classification.

All Tier 1 or Tier 2 variants in AMP classification and the Tier 3 variants that were pathogenic (P), likely pathogenic (LP), or Leaning pathogenic VUS in ACMG classification were selected as potentially important findings (PIFs). PIFs known to be therapeutic, prognostic or diagnostic biomarkers were annotated as potentially actionable findings (PAFs).

### RNA sequencing

RNA from whole blood in PaxGene tube was extracted and processed by the TruSeq Stranded Total RNA with Ribo-Zero Gold Kit (Illumina Inc., CA, USA) using 1 µg of DNAse treated RNA. Then paired-end sequencing was performed on Illumina platforms.

CLC genomics workbench 20 was used for RNA sequencing and fusion gene analyses. RNA sequencing results were used for determining the outlier expression analysis of genes harboring important/actionable variants, analyzing the fusion transcripts as well as confirmation of variants found in WES.

### Chromosome-sequencing

Vist*a™* Chromosome Sequencing-100 K an NGS based Whole Genome Sequencing was performed in BGI Clinical Laboratories to evaluate copy number variations (≥ 100 Kb).

### Cross validation of the identified variants

The identified SNVs and small Indels in WES were visually validated in the mapping read-tracks after importing WES BAM files in the CLC genomics workbench 20. Besides, the validations of identified WES variants were performed using RNASeq BAM files by confirming both variant and gene expression.

The detected CNVs in chromosome-seq data were validated by CNV analysis from WES data using CLC genomics workbench 20.

### Ethics approval and consent to participate

The study was approved by the Ethical Committee of the Iran University of Medical Sciences (Code: IR.IUMS.REC 1395.95-04-87-30235). Informed consent was obtained from all subjects and/or their legal guardian(s) for participation.

## Results

### Clinical findings

The patient (I), was a 66-year-old female presented by fever, hepatosplenomegaly, leukocytosis, anemia, thrombocytopenia. The blast level in the peripheral blood was 25%. Bone marrow aspiration and immunophenotyping confirmed the myeloid blastic crisis. The patient had a history of thyroid cancer that had been treated many years prior to the development of CML. She was taken to the hospital at the age of 63 with a primary diagnosis of chronic phase CML in terms of leukocytosis with myeloid precursors in the peripheral blood and a positive Philadelphia chromosome in karyotype.

The patient (II), a 55-year old female presented by leukocytosis, anemia, thrombocytopenia. The diagnosis of chronic phase CML was established 15 years before blastic phase at the age of 40 years. There was 36% blast in the peripheral blood. Bone marrow aspiration and immunophenotyping confirmed the myeloid blastic crisis. At blast crisis, the peripheral blood karyotype revealed a clone that was positive for both the philadelphia chromosome and isochromosome 17q, as well as a clone with paracentric inversion in 15q.

Patient (III) was a 45-year old male presented by leukocytosis, anemia and thrombocytopenia. The diagnosis of chronic phase CML was established two years before blastic phase at the age of 43 years. There was 40% blast in the peripheral blood. Bone marrow aspiration and immunophenotyping confirmed the myeloid blastic crisis.

*BCR-ABL1* fusion transcript (b13-a2) was also found in the RNA-seq fusion gene study of all patients during the blast crisis phase.

Table 1 summarizes the patients' detailed clinical and paraclinical findings at chronic phase CML diagnosis. The clinical and paraclinical findings at myeloid blast crisis are presented in the Table 2.

**Table 1:** Clinical and laboratory findings for the CML patients at diagnosis/chronic phase.

Patient no	Patient 1	Patient 2	Patient 3
Gender	Female	Female	Male
Age at diagnosis (years)	63	40	43
WBC per µL	18,500	165,200	40,000
Diff (CBC)	MyeloBlast: 2%, Granolocyte: 79%, Lymphocyte: 10%; Eosinophil: 3%; MetaMyelocyte: 3%; Band: 3%	MyeloBlast: 3%, Granolocyte: 58%, Lymphocyte: 9%; Eosinophil: 1%; MetaMyelocyte: 7%; Band: 11%; Myelocyte: 8%; ProMyelcyte: 3%	MyeloBlast: 6% Granolocyte: 38%; Lymph:17%; Eosinophil: 4%; MetaMyelocyte: 15%; Band: 3%; Basophil: 1%; Myelocyte: 14%; Monocyte: 2%
Platelet per µL	1,918,000	703,000	711,000
Hemoglobin (mg/dL)	9.6	12.5	11.6
Spleen Size	Palpable (2 cm)	Palpable (2 cm)	NA
Sokal relative risk score	3.79 (high)	0.93 (intermediate)	1.18 (intermediate)
Bone Marrow Pathology	High and compact cellularity; Marked myeloid hyperplasia; Giant and lobulated Megakaryocytes with marked hyperplasia; Plasmocyte:3%; Lymphoid: 4%; Normal Iron storage	The marrow is 100% cellular. The hematopoietic populations are replaced by atypical granulocytic series, composed of prominent segmented neutrophils mixed with immature forms including myelocytes and promyelocytes. Myeloblasts in sheets are about 60% of populations. The marrow framework is fibrotic in occasional focal areas	NA
*BCR*-*ABL1* RT-PCR	Positive	Positive	Positive
*ABL1* kinase domain Mutation analysis (by PCR-sanger sequencing)	p.F359V mutation was detected (35 months after diagnosis)	p.G250E mutation was detected (74 months after diagnosis) p.F359V mutation was detected (164 months after diagnosis at MBC phase)	No mutation was detected (22 months after diagnosis at MBC phase)
Probable Mechanism of TKI Resistance	*ABL1* kinase domain p.F359V Mutation causes resistance to imatinib and nilotinib	*ABL1* kinase domain p.G250E Mutation causes resistance to imatinib	Additional cytogenetic abnormalities (according to the chromosome-seq data in Table 2)

*Dx* diagnosis, *NA* not available, *RT-PCR* reverse transcriptase PCR, *TKI* tyrosine kinase inhibitor.

**Table 2:** Clinical and laboratory findings for the CML patients in MBC phase.

Patient no	Patient 1	Patient 2	Patient 3
Age of MBC (years)	66	55	45
Age of death	67 (16 days after MBC Dx)	55 (6 month after MBC Dx)	45 (3 month after MBC Dx)
WBC per µL	55,350	93,520	16,310
Diff (CBC)	Blast: 25%, Granolocyte: 55%, Lymphocyte: 20%	Blast: 36%, Granolocyte: 59%, Lymphocyte: 5%	Blast: 40% Granolocyte: 50%; Lymph:10%
Platelet per µL	53,000	41,000	11,000
Hemoglobin (mg/dL)	7.4	7.8	6.4
Spleen Size	144*63 mm	30 mm below the left costal margin	192*70 mm; huge splenomegaly
Bone Marrow Pathology	Myeloid hyperplasia with large myeloblast	Bone trabeculea and marrow spaces with about 90% cellularity composed of sheets of blast like cells	Hyper cellular marrow with fibrosis- Blast: 40% 30–40% of cells were positive for CD34
Karyotype	46,XX, der(9)t(9;22)(q34;q11.2)t(9;22)(q12;p13), der(22)t(9;22)(q34;q11.2), der(22)t(9;22)(q12; p13)[50]	46,XX, t(9;22)(q34;q11.2),i(17)(q10)[17] /46,sl,inv(15)(q21,q25)[33]	NA
Chromosome seq [GRCh37/hg19]	No chromosome aneuploidy and no pathogenic variation of chromosome microdeletion or microduplication was detected	46,XX,del(15q26.3).seq(99,294,139–100,356,602) × 1; del(17p11.2p13.3).seq(1–18,903,731) × 1; dup(17p11.2q25.3).seq(18,921,492–81,170,888) × 3	46,XY,del(7p14.3p21.1).seq (20,655,612–29,695,336) × 1; del(7p11.2p14.3).seq(33,201,686–57,488,614) × 1; del(7q11.23q21.11).seq(76,745,520–85,788,290) × 1
Quantitative *BCR*-ABL (PB)	24.1%	62.4%	58%
Immunophenotyping	Positive for Myeloid markers: (MPO, CD33), Negative for Lymphoid markers (CD3,CD19,CD20) and CD34 Positive	Positive for Myeloid markers: (MPO, CD33, CD117), Negative for Lymphoid markers (CD3,CD19,CD20) and CD34 Positive	Positive for Myeloid markers: (MPO), Negative for Lymphoid markers (CD10,CD19(1),CD20(1)) and CD34 Positive
Specific Therapeutic Management at MBC	Cytarabine; Arsenic (discontinued after two days due to cardiotoxicity); Dasatinib	Hydroxyurea; Cytarabine; Nilotinib; Dasatinib	Hydroxyurae; Imatinib; Cytarabine; Cludarabin; Danarubicin; Mitoxantrone; Nilotinib
HSCT	[HSCT has been suggested but not performed]	Incomplete [HLA typing was conducted but appropriate donor was not identified]	Incomplete [HLA typing was conducted but HSCT was not performed due to the rapid disease progression and death].

*Dx* diagnosis, *NA* not available, *MBC* myeloid blast crisis, *HSCT* hematopoietic stem cell transplantation.

### Genetic findings

**Figure 1:**
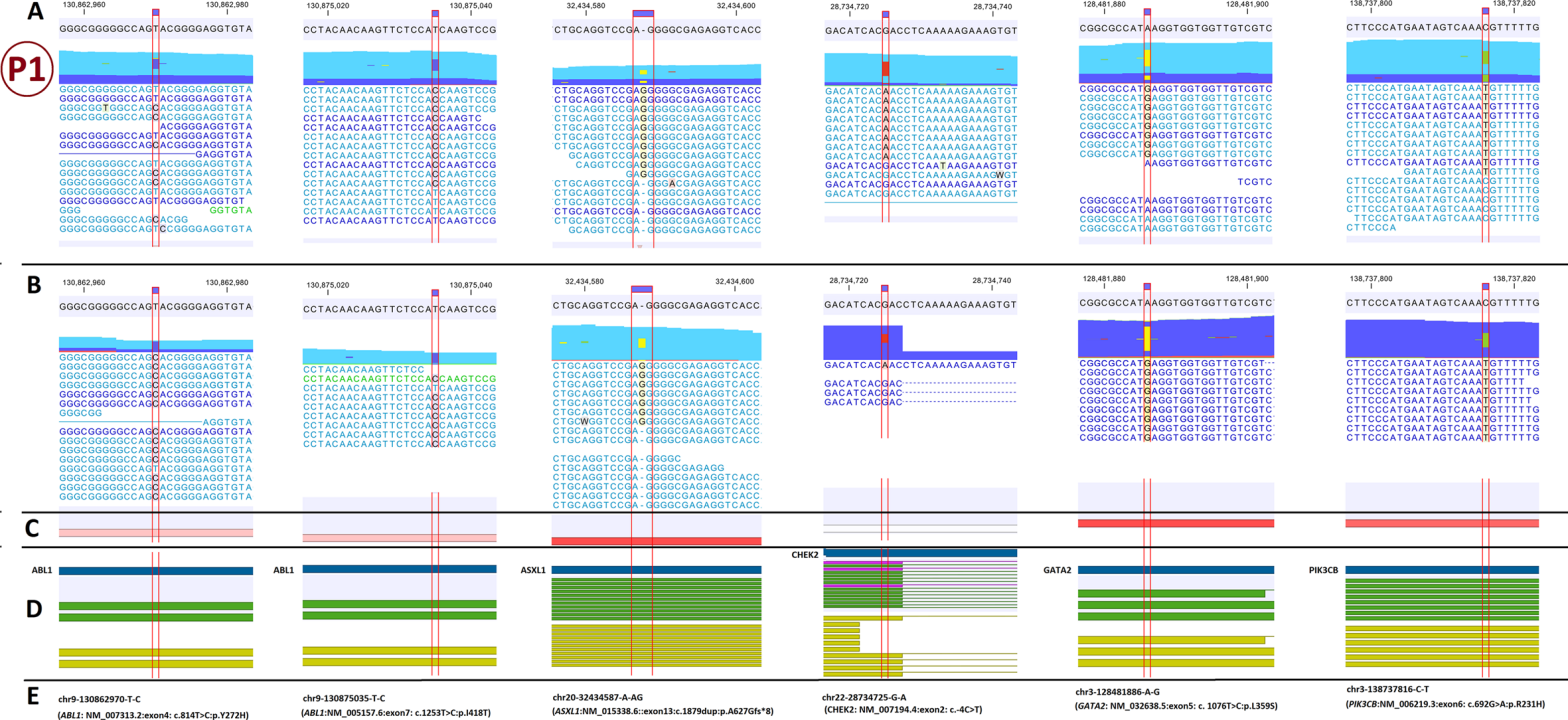
The paired-end mapping read-track displaying reads and detected variants in the first patient. The potentially important findings from WES data analysis is presented in the CLC genomics workbench software. (**A**) hg38 sequence track with WES mapped reads track; (**B**) hg38 sequence track with RNA-Seq mapped reads track; (**C**) Gene expression track; (**D**) gene, mRNA and CDS tracks; (**E**) the variant genomic coordinate on hg38 and the variant name. P1: patient No.1.

**Figure 2:**
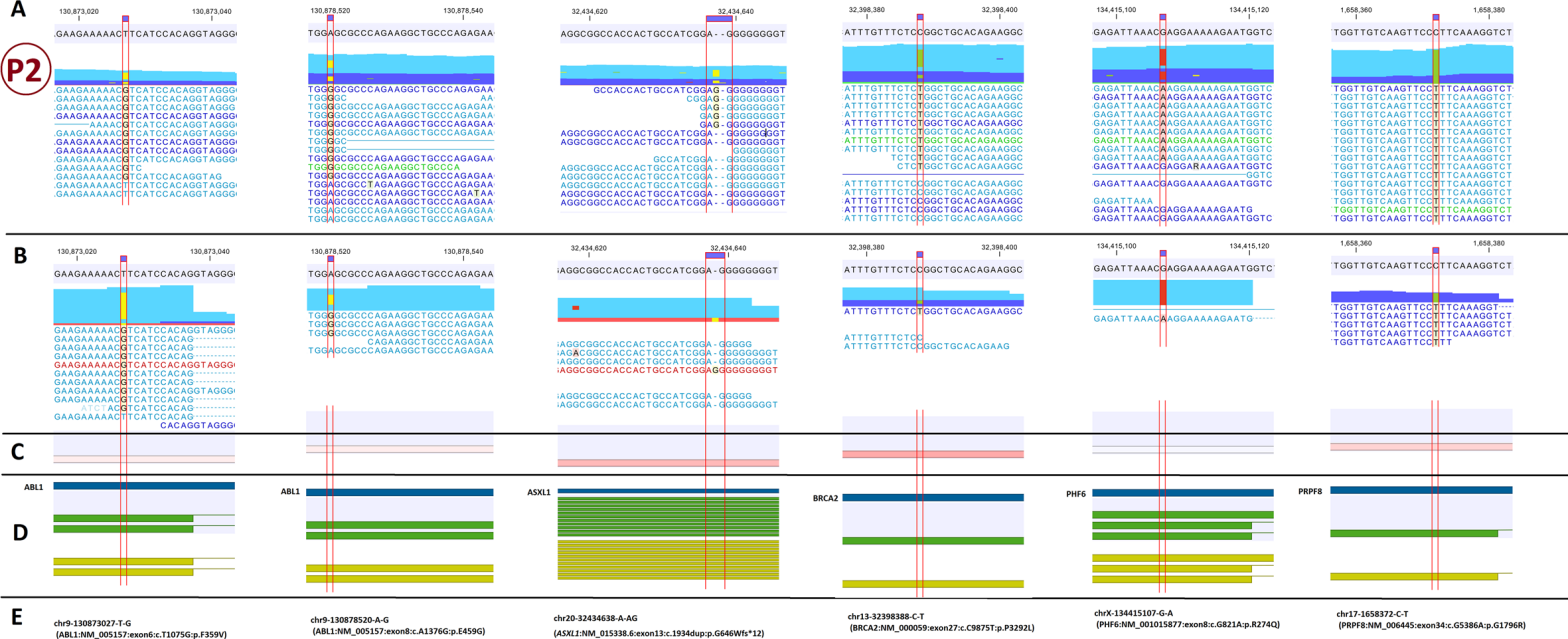
The paired-end mapping read-track displaying reads and detected variants in the second patient. The potentially important findings from WES data analysis is presented in the CLC genomics workbench software. (**A**) hg38 sequence track with WES mapped reads track; (**B**) hg38 sequence track with RNA-Seq mapped reads track; (**C**) Gene expression track; (**D**) gene, mRNA and CDS tracks; (**E**) the variant genomic coordinate on hg38 and the variant name. P2: patient No.2.

**Figure 3:**
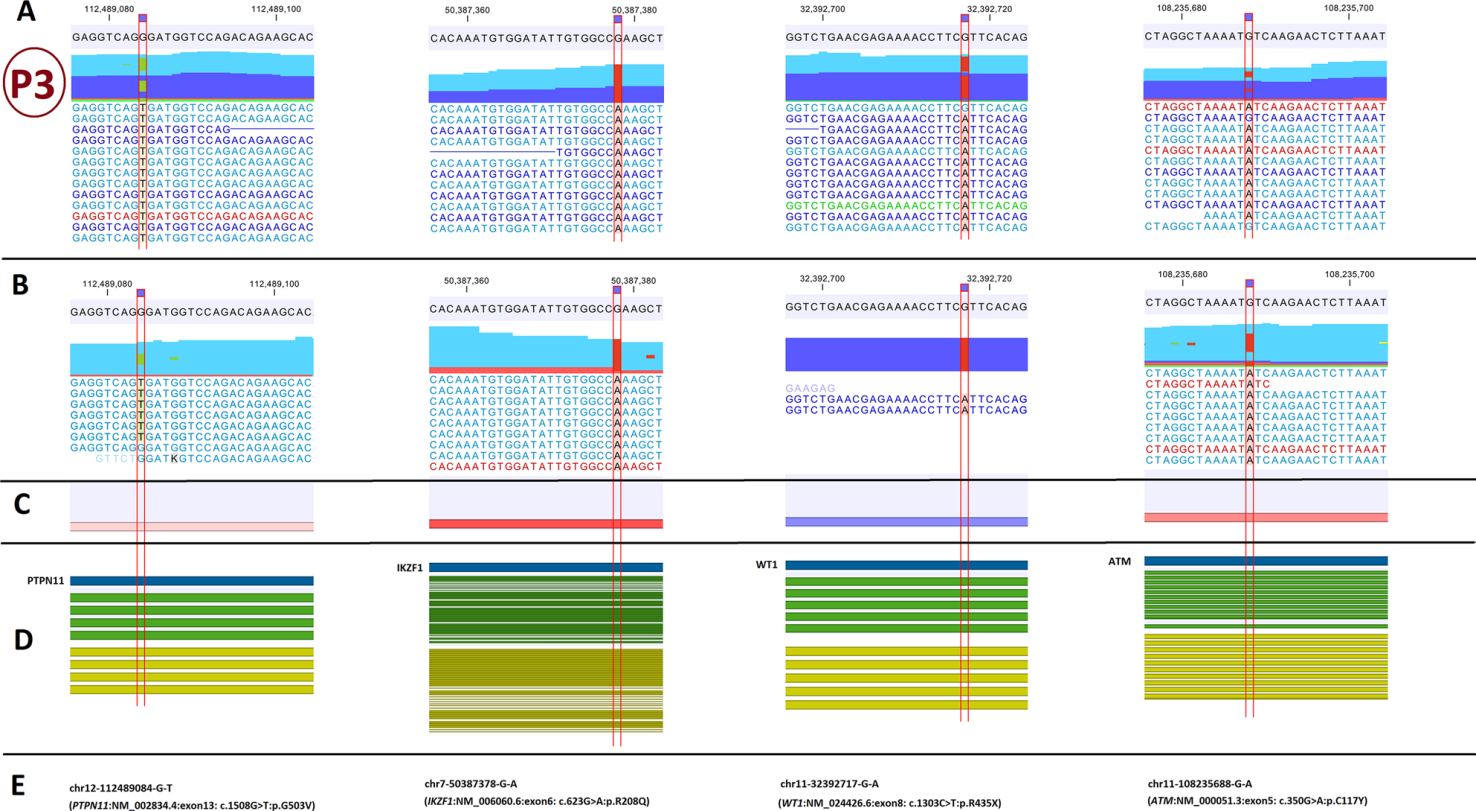
The paired-end mapping read-track displaying reads and detected variants in the third patient. The potentially important findings from WES data analysis is presented in the CLC genomics workbench software. (**A**) hg38 sequence track with WES mapped reads track; (**B**) hg38 sequence track with RNA-Seq mapped reads track; (**C**) Gene expression track; (**D**) gene, mRNA and CDS tracks; (**E**) the variant genomic coordinate on hg38 and the variant name. P3: patient No.3.

WES analysis identified sixteen PIFs (Figs. 1, 2 and 3) in which *ABL1*-Y272H, *ASXL1*-A627Gfs*8, *GATA2*-L359S, *ABL1*-F359V, *ASXL1*-G646Wfs*12, *PTPN11*-G503V and *IKZF1*-R208Q variants were classified as PAFs. The *ABL1*-I418T, *PIK3CB*-R231H, *CHEK2*:c.-4C > T, *BRCA2*-P3292L, *ABL1*-E459G, *PRPF8*-G1796R, *PHF6*-R274Q, *WT1*-R435X and *ATM*-C117Y variants were potentially important cancer variants which were not actionable (Table 3).

**Table 3:** Potentially actionable (PAF) and potentially important (PIF) findings in WES analysis of BC-CML patients.

Patient	Gene: mRNA Accession#:Exon#: Variant	CancerVar Score	Leukemogenic Class	ACMG/AMP/CGI classification	CADD phred	Biomarker Type	DP (VAF)	PAF/PIF
P1	*ASXL1*:NM_015338.6::exon13:c.1879dup:p.A627Gfs*8	7	III	P/Tier1/Dv	33	T-D-P	90 (34.4%)	PAF
P1	*ABL1*: NM_007313.2:exon4: c. 814T > C:p.Y272H	8	I	P/Tier2/kDv	28.2	T	125 (43.2%)	PAF
P1	*GATA2*: NM_032638.5:exon5: c. 1076T > C:p.L359S	9	II	LP/Tier2/Dv	29.6	T-P	123 (50.4%)	PAF
P1	*ABL1*:NM_005157.6:exon7: c.1253T > C:p.I418T	4	I	LP/Tier3/Dv	25.3	T	88 (45.4%)	PIF
P1	*PIK3CB*:NM_006219.3:exon6: c.692G > A:p.R231H	8	I	VUS/Tier2/Dv	34	T	120 (45%)	PIF^$^
P1	*CHEK2*:NM_007194.4:exon2: c.-4C > T	2	IV	VUS-P/Tier3/Ps	3.46		66 (51.2%)	PIF
P2	*ABL1*:NM_005157:exon6: c.1075T > G:p.F359V	8	I	P/Tier1/kDv	32	T	40 (55%)	PAF
P2	*ASXL1*:NM_015338.6:exon13:c.1934dup:p.G646Wfs*12	9	III	P/Tier2/Dv	33	T-D-P	92 (45.6%)	PAF
P2	*BRCA2*:NM_000059.3:exon27: c.9875C > T:p.P3292L	9	IV	LB/Tier2/Dv	33	T-P	55 (50.9%)	PIF
P2	*ABL1*:NM_005157:exon8: c.1376A > G:p.E459G	7	I	LP/Tier3/kDv	33	T	94 (41.4%)	PIF
P2	*PRPF8*:NM_006445.4:exon34: c.5386G > A:p.G1796R	6	V	VUS-P/Tier3/Dv	32	T	61 (96.7%)	PIF^$^
P2	*PHF6*:NM_001015877.2:exon8: c.821G > A:p.R274Q	7	II	LP/Tier3/Dv	35	T	45 (66.6%)	PIF^$^
P3	*PTPN11*:NM_002834.4:exon13: c.1508G > T:p.G503V	10	I	P/Tier1/kDv	32	D-P	94 (52%)	PAF
P3	*IKZF1*:NM_006060.6:exon6: c.623G > A:p.R208Q	8	II	LP/Tier2/Ps	19.92	P	39 (100%)	PAF^$^
P3	*WT1*:NM_024426.6:exon8: c.1303C > T:p.R435X	7	II	P/Tier3/Ps	40	P	56 (82%)	PIF
P3	*ATM*:NM_000051.3:exon5: c.350G > A:p.C117Y	8	IV	VUS-P/Tier2/Dv	29.3	T-P	52 (30.7%)	PIF^$^

*P* pathogenic, *LP* likely pathogenic, *VUS-B* variant of uncertain significance leaning benign, *VUS-P* variant of uncertain significance leaning pathogenic, *B* benign, *LB* likely benign, *TSG* tumor suppressor gene, *OG* oncogene, *Dv* driver, *kDv* known driver, *Ps* passenger.

Biomarker type: T = Therapeutic; D = Diagnostic; P = Prognostic; DP: Depth of coverage; VAF: Variant Allele Fraction.

Leukemogenic Class: I (Signaling); II (Transcription Factor), III (Epigenetic Regulator); IV (TSG); V (Spliceosome).

^$^Novel variants in CML.

Npa: not protein affecting.

### Genetic findings in the patient no.1

The patient was a *BCR*-*ABL1* positive CML case in the myeloid blast crisis phase. She had abnormal karyotype with Philadelphia chromosome. In chromosome-seq there were no specific copy number alterations.

In WES she had six PIFs which contributed in all classes of leukemogenic genes (Fig. 1) except splicing components (class V). The p.Y272H variant on *ABL1* (class I), p.A627Gfs*8 on *ASXL1* (class III) and p.L359S on *GATA2* (class II) were PAFs.

### Genetic findings in the patient no.2

The patient was a *BCR*-*ABL1* positive CML case in the myeloid blast crisis phase. She had abnormal complex karyotype with Philadelphia chromosome and isochromosome 17q in one clone and paracentric inversion in 15q in the another stemline (46,XX, t(9;22)(q34;q11.2),i(17)(q10)[17] /46,sl,inv(15)(q21,q25)[33]). In chromosome-seq (Fig. S1) there were deletions in 15q and 17p, and duplication in 17q (46,XX,del(15q26.3); del(17p11.2p13.3); dup(17p11.2q25.3)). The *TP53* gene is a class II leukemogenic gene (transcription factor) located in 17p13.1 which is deleted in this patient.

In WES she had six PIFs that contribute in all classes of leukemogenic genes (Fig. 2). Considering the *TP53* involvement by 17p deletion, this patient has seven PIFs. The p.F359V variant in *ABL1* (class I), p.G646Wfs*12 in *ASXL1* (class III) and *TP53* (class IV) deletion were PAFs.

### Genetic findings in the patient no.3

The patient was a *BCR*-*ABL1* positive myeloid blast crisis CML. In chromosome-seq (Fig. S1) he had several deletions in 7p and 7q (46,XY,del(7p14.3p21.1); del(7p11.2p14.3); del(7q11.23q21.11)). The *IKZF1* (7p12.2), *EZH2* (7q35-q36) and *CUX1* (7q22) are important leukemogenic genes which are located in the deletion regions. *EZH2* gene is an epigenetic regulator gene (H3K27 methyltransferase; class III) and *CUX1* is a transcription factor (class II) regulating *TP53* and *ATM*.

He also had four PIFs in WES (Fig. 3). In terms of the pathogenic deletions in the short and long arms of chromosome 7 affecting *EZH2* and *CUX1,* this patient has six PIFs which involve four classes of leukemogenic genes. *PTPN11*:p.G507V (class I) and *IKZF1*:p.R65Q variants (class II) were PAFs. The *WT1*:p.R435X (class II) and *ATM*:p.C117Y (class IV) variants were not PAFs.

*IKZF1* variant (p.R65Q) in combination with del(7p11.2p14.3) may affect *IKZF1* gene product as an important transcription factor in myeloid malignancies.

## Discussion

The ability to analyze and model the leukemogenesis process may aid in the effective management and treatment of hematologic malignancies. According to the proposed “Slot machine” model of leukemogenesis by Murati et al.^4^, progression from chronic phase into secondary acute myeloid leukemia (blast crisis) can be modeled by the combination of at least four steps that one classes of leukemogenic genes is affected in each step.

**Figure 4:**
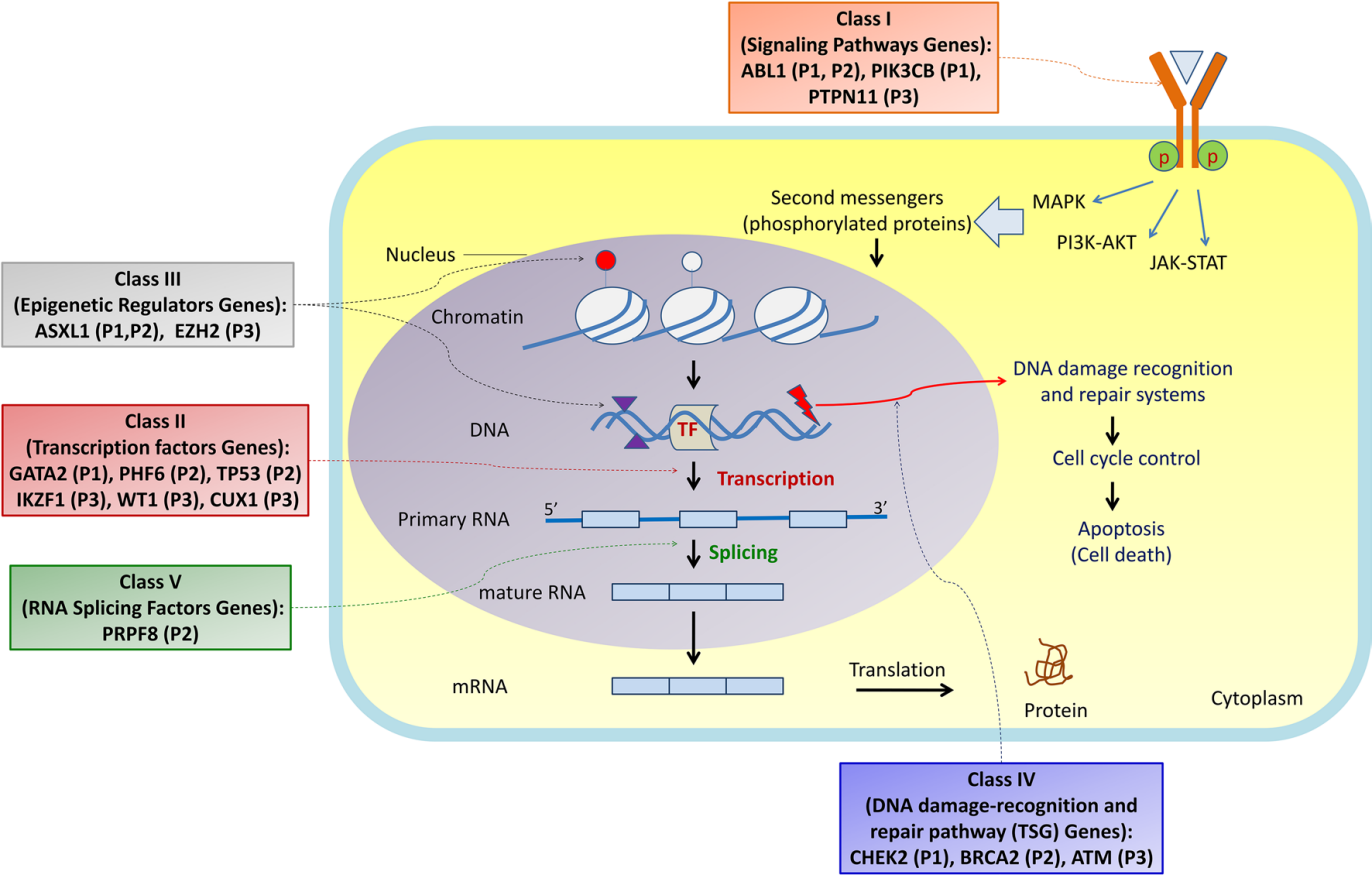
The detected SNV and Indel variants in WES related to leukemogenic gene classes. The classes of leukemogenic genes includ signaling pathway genes (class I), transcription factors (class II), epigenetic regulators (class III), tumor suppressor and DNA repair genes (class IV) and components of the spliceosome (class V).

Using integrated genomic sequencing to assess the genes affected by SNVs, Indels and CNVs, in the present study all MBC-CML patients had at least five leukemogenic genes involved. The detected SNV and Indel variants in WES represent all classes of leukemogenic genes (Fig. 4) including signaling pathway gene (*ABL1*-Y272H, *ABL1*-I418T, *ABL1*-F359V, *ABL1*-E459G, *PIK3CB*-R231H, *PTPN11*-G503V), transcription factors (*GATA2*-L359S, *PHF6*-R274Q, *IKZF1*-R208Q, *WT1*-R435X), epigenetic regulators (*ASXL1*-A627Gfs*8, *ASXL1*-G646Wfs*12), tumor suppressor and DNA repair genes (*BRCA2*-P3292L, *ATM*-C117Y, *CHEK2*:c.-4C > T) and components of the spliceosome (*PRPF8*-G1796R).

The detected variants in different signaling pathway related genes including *ABL1*, *PIK3CB* and *PTPN11* in MBC-CML patients may affect ErbB, PI3K-Akt, Ras-MAPK cascade and JAK-STAT signaling pathways^15–17^. *ABL1* was involved in the first and second patients. Four important missense variants were detected in *ABL1* (p.Y272H, p.I418T, p.F359V, p.E459G). All variants were located in the tyrosine kinase domain of *ABL1* and were previously reported in CML (COSM12576, COSM12605, COSM1460549)^18^. Mutations in the ABL1 kinase domain, in particular, have been identified in CML patients in the blastic phase^19^. Occurrence of 2 or more mutations in *BCR*–*ABL1* fusion gene following TKI therapy has been reported in CML progression^20^.

*PIK3CB*-R231H is an important missense variant in the Ras Binding domain (RB domain). This deleterious variant in *PIK3CB* gene has not been reported in the hematological malignancies including CML (https://www.mycancergenome.org/content/gene/pik3cb/). This gene plays a critical role in solid tumor development^16,21^.

The third patient’s *PTPN11*-G507V variant is located in the Phosphatase (PH) domain. Although PTPN11 mutations have been documented in blastic phase CML, the p.G507V alteration is a novel variant in CML and has only been identified in other myeloid malignancies such as JMML, AML and MDS. (COSM14271).

There were several important variants in different transcription factor genes including *GATA2*, *PHF6*, *WT1* and *IKZF1* in MBC-CML patients. In addition, *TP53*, *IKZF1* and *CUX1* genes were affected by chromosomal deletions/CNVs. *GATA2*-L359S variant in the first patient is located on the zinc finger GATA domain. It has been indicated that *GATA2* germline mutations strongly predispose patients to leukemia^22^. GATA2 mutations were described in MBC-CML; however p.L359S variant has not been previously reported in blastic CML. p.L359S variant was suggested as a predisposing germline likely pathogenic variant in MDS/AML^23^.

*PHF6*-R274Q variant in patient No.2 is located in the PHD-like zinc binding domain. Mutations in *PHF6* also have been reported in the blastic phase CML while p.R274Q variant is a novel mutation in CML which only reported in Early T-cell precursor (ETP) acute lymphoblastic leukemia (COSM306061).

Biallelic involvement of *IKZF1* was observed in the third patient. The detected missense variant was located in the C2H2 zinc finger domain of the *IKZF1* (p.R65Q). Because of the 7p deletion, this variant showed a high variant allele fraction (VAF = 100%) in WES analysis. *IKZF1* mutations had been found in both chronic and blastic phase CML^24^.The patient with *IKZF1* mutation also had a mutation in *PTPN11*. It has been shown that in AML patients, mutations in *IKZF1* and *PTPN11* are associated with aggressive clinical course and primary resistant to chemotherapy^25^. *CUX1* gene involvement was detected in the third patient in the cytogenetic studies in terms of 7q deletion. *CUX1* is a transcription factor regulating *TP53* and *ATM*^26^.

17p deletion in *TP53* gene was established by cytogenetic analysis in the second patient. *TP53* is a transcription factor located in 17p13.1. *TP53* regulate cell cycle, DNA repair and apoptosis. It was observed that 45% of blast phase MPN (MPN-BP) patients have a *TP53*-related defect such as *TP53* gene mutations or haploinsufficiency^27^. Involvement of *TP53* has been described in both chronic and blastic phases of CML^28,29^.

Additional chromosomal abnormalities (ACAs) have been discovered in the second and the third patients. Patients with high-risk ACA, such as i(17q) and -7/7q-, are known to have a poor response to TKIs and a greater risk of disease progression. -7/7q- are less common in BC than i(17q), although they have a greater unfavorable influence on prognosis^30^. The patient number 3 with multiple deletions in the chromosome 7 has shorter time to MBC.

*WT1-R435X* is another significant nonsense variant discovered in the third patient. *WT1* is a tumor suppressor gene which has known function as a transcriptional regulator^31^. This mutation causes loss of function in *WT1* by the activation of NMD (Nonsense mediated decay) mechanism. Mutations in *WT1* were reported in the blastic phase CML^32^, Wilms tumor and desmoplastic small round cell tumor (COSM21401). Pathogenic variants in this gene are correlated with poor prognosis in the patients with myelodysplastic syndromes^33^. The p.R435X germline mutation in *WT1* has been classified as pathogenic which is associated to Wilms tumor in the clinvar (RCV000003671).

Two frameshift single nucleotide deletions were identified in *ASXL1* (A627Gfs*8, G646Wfs*12). *ASXL1* mutations are more frequent in secondary AML and may contribute to the development of CML to AML. Moreover, *ASXL1* mutation might be associated with an aggressive phenotype in myeloid malignancies^4^.

It has been shown that mutations in ABL1 kinase domain, *ASXL1, IKZF1, TP53* are common in CML patients who develop BC^12,30,34^. *ASXL1* mutation is the most frequent mutation in poor outcome patients. In addition, individuals with an *ASXL1* mutation had a longer median time to develop BC^12^. In the current study, the first and the second patients with *ASXL1* mutation had longer time to MBC than the third patient.

*EZH2* gene deletion was established by chromosome-seq in the third patient. *EZH2* gene is an epigenetic regulator gene (H3K27 methyltransferase). Loss-of-function mutations in *EZH2* have been reported in primary myelofibrosis (PMF), MDS and MDS/MPN overlap syndromes^27^.

Several important variants have been documented in the tumor suppressor genes specially the genes involved in the DNA damage-recognition and DNA repair pathways including *CHEK2*, *ATM* and *BRCA2*. The *CHEK2* c.-4C > T variant is an important Tier3 variant. *CHEK2* has a role in DNA damage-recognition and repair, cell cycle and P53 signaling pathway. The c.-4C > T variant is located in the kozak sequence which may affect translation^35^. This variant has been reported in clinvar as VUS for hereditary cancer-predisposing syndrome (RCV000131287).

*ATM*-C117Y variant is a Tier2 missense variant. *ATM* is involved in homologous recombination, cell cycle and P53 signaling pathway^36^. *ATM* has a key role in the DNA damage-response pathway and loss of function mutations in *ATM* may promote acceleration of the blast crisis in CML^37^. The ATM-CHEK2-p53 axis has been shown to play an important role against cancer initiation by inducing apoptosis, cell cycle arrest or senescence in the cancer cells^27^.

*BRCA2*-P3292L variant is a missense Tier2 variant. *BRCA2* is a tumor suppressor gene which contributes to homologous recombination^38^. It has been indicated that 20% of the patients with Therapy-Related Myeloid Neoplasms (t-MN) among breast cancer survivors have a germline mutation in the *BRCA1/2, CHEK2, TP53,* or *PALB2* genes, which are important in DNA repair pathways^27^.

*PRPF8* was the only RNA splicing factor gene with a significant variant*.* It is a core component of U2-type and U12-type spliceosome complexes^39^. *PRPF8*-G1796R variant in the second patient is a missense Tier3 variant. This variant has been classified as driver mutation and has not been previously reported.

The self-renewal, differentiation and proliferation are three main processes which are impaired in malignancies. In the current study, the integrated genomic sequencing detected important variants in twelve different cancer genes. Mutations in signaling genes may induce proliferation of malignant cells while mutations in the epigenetic regulators (ERs) or tumor suppressor genes (TSG) may promote self-renewal in the progenitor cells and transcription factors mutations may affect differentiation. Mutations in the spliceosome component may also involve differentiation and self-renewal of progenitor cells^40^. Therefore, the alterations in *ABL1*, *PTPN11* and *PIK3CB* signaling molecules may potentiate the proliferation process in MBC-CML. Variants in the *CHEK2*, *BRCA2* and *ATM* tumor suppressor genes, as well as the *ASXL1* and *EZH2* Epigenetic Regulators, may support the self-renewal of the proliferating progenitors. Variants in the *GATA2*, *PHF6*, *WT1*, *IKZF1* and *CUX1* transcription factors may operate as differentiation blockers. Finally, the variant in the *PRPF8* as spliceosome components may act as differentiation blocker or may cause self-renewal of the proliferating progenitor.

Finally, a recent integrated model of blast crisis^41^ showed that one of the most important mechanism associated to the BC progression is the epigenetic reprogramming related to the abnormal Polycomb Repressive Complex (PRC) function. The components of the PRC complex as an epigenetic complex, repress the expression of certain genes including leukemogenic *HOXA* cluster. On the other hand, specific gene products interact with PRC components to add/remove repressive marks or recruitment of PRC components to distinct loci. Based on recent studies on BC genome and transcriptome, it has been shown that the components of the PRC complex have mutated or altered in expression respectively. These changes overally affect the gene expression signature of BC cells. *ASXL1* gene products interact with PRC2 components including *EZH2*. *IKZF1* also recruits PRC2 to the target gene loci. According to this model, in the first and second patients the frameshift mutations in the *ASXL1* may have a critical role in BC progression. In the third patient the large deletions in the chromosome 7 which affect *IKZF1* (7p12.2) and *EZH2* (7q35-q36) may accelerate BC.

**Figure 5:**
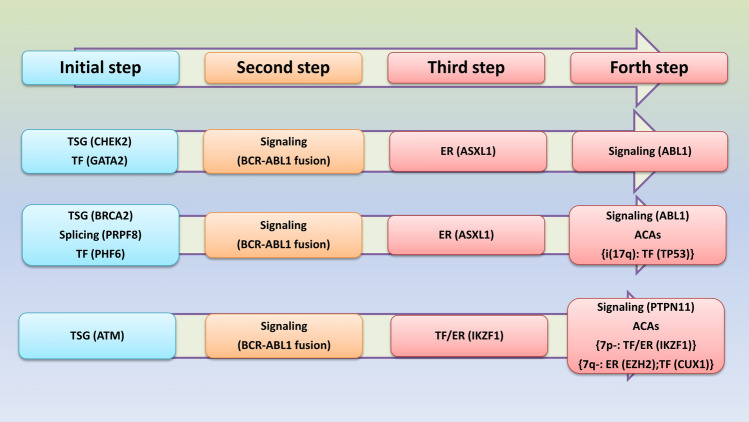
A suggested model of leukemogenesis. In this model the initial step is the presence of mildly-deleterious variants which may affect the tumor suppressor genes (TSG), transcription factors (TF) and/or components of splisosome and increase susceptibility to cancer. In the second and third steps, the major genetic findings are *BCR-ABL1* fusion gene and mutation in the epigenetic regulator (ER)/transcription factor (TF) genes. The final step in the accelerated and blast crisis phase may be started by the mutations in the signaling molecules and/or additional chromosomal abnormalities (ACAs).

A suggested model of leukemogenesis for the hierarchy of events is presented in the Fig. 5. In this model the initial step is the presence of mildly-deleterious variants (VUS-P) mainly in the tumor suppressor genes (TSG)/DNA damage repair (DDR) genes, transcription factors (TF) and/or components of splisosome. These variants may increase the individual’s susceptibility and predisposition to malignancies. The BCR-ABL1 fusion gene and mutations in the epigenetic regulator (ER)/transcription factor (TF) genes, which mainly influence the function of PRC, are the key genetic discoveries in the second and third steps. The final step in the accelerated and blast crisis phase may be started by the mutations in signaling molecules and/or additional chromosomal abnormalities (ACAs). *ABL1* mutations specifically in the kinase domain lead to TKI resistance and delay in TKI switching may affect prognosis and survival by blast crisis. ACAs which involve leukemogenic genes (such as ER, TF or TSG) also may exacerbate the clinical prognosis and enter the disease to the blastic phase.

Although mutations in *ASXL1* or *IKZF1* genes may play a key role in BC, they are more often found at the time of diagnosis in the chronic phase, in individuals with poor prognosis. Therefore, mutations in oncogenes such as *ABL1* and *PTPN11* may act as an accelerator.

In this study, samples related to the different phases of disease progression were not available. Future studies using genomic sequencing in different serial time points may allow detecting the sequence of occurrences related to the disease progression. Furthermore, recent advances in single cell sequencing may help to more accurately clarify the disease mechanisms.

## Conclusions

The integrated genomic sequencing in this study effectively identified large spectrum of important and actionable variants from SNVs to CNVs. Mildly-deleterious passenger variants mainly in DNA damage repair (DDR) genes may increase the individual’s predisposition to cancer. *BCR-ABL1* fusion, mutations in signaling and epigenetic regulator genes along with ACAs may act as cancer drivers. Epigenetic reprogramming caused by *ASXL1* or *IKZF1* mutations seems to be one of the most critical mechanisms linked with BC development. *ABL1* domain mutations, mutations in other signaling genes and high risk ACAs may promote CML to BC.

Using this strategy we have identified several actionable findings which may improve targeted therapies. These findings may help better understanding the underlying pathophysiological mechanisms and may provide further insights into MBC-CML genetic basis in future. Further studies will shed light on the clinical usefulness of the integrated genomic analysis.

## Supplementary Information

Supplementary Information 1.

Supplementary Information 2.

## Data Availability

The datasets generated and/or analyzed during the current study are available in the ENA https://www.ebi.ac.uk/ena/browser/view/PRJEB51084.

## Abbreviations

AP: Accelerated phase
DP: Depth of coverage
ACMG: American college of medical genetics and genomics
ASCO: American society of clinical oncology
AMP: Association for molecular pathology
B: Benign
BMA: Bone marrow aspiration
CML: Chronic myeloid leukemia
CGI: Cancer genome interpreter
CAP: College of American pathologists
CNV: Copy number variation
Dx: Diagnosis
Dv: Driver
ER: Epigenetic regulators
GATK: Genome analysis tool kit
kDv: Known driver
LPC: Leukemia progenitor cell
LSC: Leukemia stem cell
LB: Likely benign
LP: Likely pathogenic
MDS: Myelodysplastic syndrome
MBC: Myeloid blast crisis
MPS: Massively parallel sequencing
MPNs: Myeloproliferative neoplasms
NGS: Next generation sequencing
Npa: Not protein affecting
Ps: Passenger
P: Pathogenic
PB: Peripheral blood
PIF: Potentially important finding
PAF: Potentially actionable finding
RT-PCR: Reverse transcriptase-polymerase chain reaction
SNV: Single nucleotide variant
TF: Transcription factor
TSG: Tumor suppressor gene
VUS: Variant of uncertain significance
VUS-B: Variant of uncertain significance leaning benign
VUS-P: Variant of uncertain significance leaning pathogenic
VAF: Variant allele fraction
WES: Whole exome sequencing
