# Author Correction: Integrated genomic sequencing in myeloid blast crisis chronic myeloid leukemia (MBC-CML), identified potentially important findings in the context of leukemogenesis model

**DOI:** 10.1038/s41598-023-27872-1

**Published:** 2023-01-13

**Authors:** Golnaz Ensieh Kazemi‑Sefat, Mohammad Keramatipour, Mohammad Vaezi, Seyed Mohsen Razavi, Kaveh Kavousi, Amin Talebi, Shahrbano Rostami, Marjan Yaghmaie, Bahram Chahardouli, Saeed Talebi, Kazem Mousavizadeh

**Affiliations:** 1 Department of Molecular Medicine, Faculty of Advanced Technologies in Medicine, Iran University of Medical Sciences, Tehran, Iran; 2 Cellular and Molecular Research Center, Iran University of Medical Sciences, Tehran, Iran; 3 Department of Medical Genetics, School of Medicine, Tehran University of Medical Sciences, Tehran, Iran; 4 Hematology, Oncology and Stem Cell Transplantation Research CenterShariati Hospital, Tehran University of Medical Sciences, Tehran, Iran; 5 Oncopathology Research Center, School of Medicine, Iran University of Medical Sciences, Tehran, Iran; 6 Laboratory of Complex Biological Systems and Bioinformatics (CBB), Department of Bioinformatics, Institute of Biochemistry and Biophysics (IBB), University of Tehran, Tehran, Iran; 7 Department of Medical Genetics, Faculty of Medicine, Mashhad University of Medical Sciences, Mashhad, Iran; 8 Department of Medical Genetics, School of Medicine, Iran University of Medical Sciences, Tehran, Iran; 9 Department of Pharmacology, School of Medicine, Iran University of Medical Sciences, Tehran, Iran

Correction to: *Scientific Reports* 10.1038/s41598-022-17232-w, published online 27 July 2022

The original version of this Article contained errors in the spelling of the authors Shahrbano Rostami and Marjan Yaghmaie which were incorrectly given as Shahrbanoo Rostami and Marjan Yaghmaei respectively.

The original Article has been corrected.

