# Supplementary figures and images for: Integrated genomic sequencing in myeloid blast crisis chronic myeloid leukemia (MBC-CML), identified potentially important findings in the context of leukemogenesis model
Source: Sci Rep. 2022 Jul 27;12:12816. doi: 10.1038/s41598-022-17232-w (PMC9329277; doi:10.1038/s41598-022-17232-w)

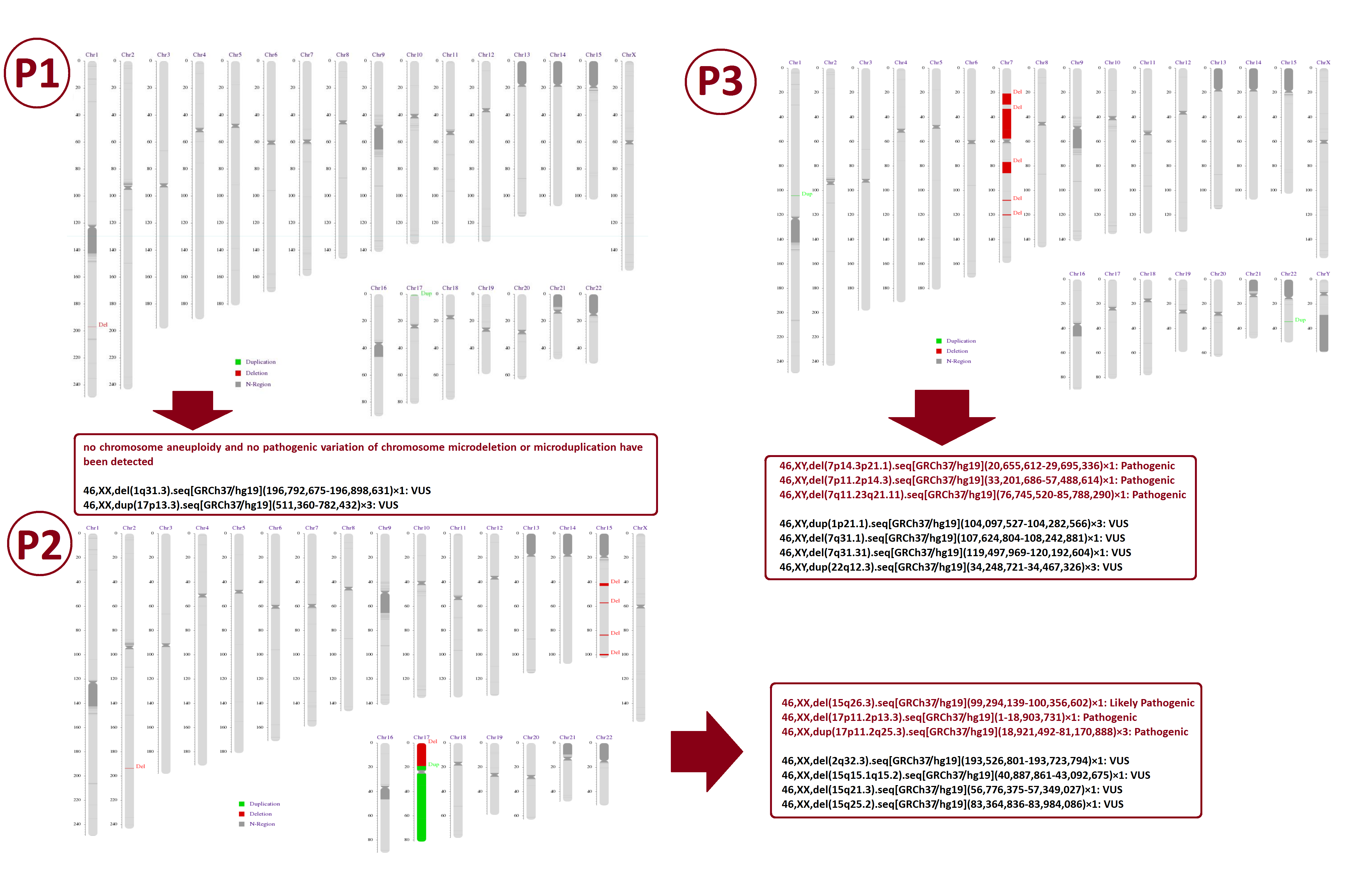

Supplement: Supplementary file 1 — Supplementary Information 1. [file 41598_2022_17232_MOESM1_ESM.tif]
