# Supplementary material for: Integrated genomic sequencing in myeloid blast crisis chronic myeloid leukemia (MBC-CML), identified potentially important findings in the context of leukemogenesis model
Source: Sci Rep. 2022 Jul 27;12:12816. doi: 10.1038/s41598-022-17232-w (PMC9329277; doi:10.1038/s41598-022-17232-w)
Supplement: Supplementary file 2 — Supplementary Information 2. [file 41598_2022_17232_MOESM2_ESM.docx]

**Table S1:** The summary report of data analysis metrics from WES

| Analytical characteristics | PATIENTS | | |
| --- | --- | --- | --- |
|  | **Patient 1** | **Patient 2** | **Patient 3** |
| Total number of reads | 53218156 | 81761136 | 60367774 |
| Read length (bp) | 151 | 101 | 151 |
| Average read length (Gbp) | 8.03 | 8.26 | 8.4 |
| Target Region (Mbp) | 38.2 | 38.2 | 33.16 |
| Capturing Kit | Sureselect V6 | Sureselect V6 | TWIST |
| % Bases QV>30 | 94.35 | 95.8 | 98.4 |
| % Initial Mappable Reads | 99.72 | 99.76 | 99.59 |
| % Minimum coverage of target regions (for depth 1X, 5X and 10X) | 98; 97; 95.8 | (98.1;97.6;96.6) | (98.7;98.5;98.4) |
| % of duplicate reads (pre-alignment) | 83.71 | 83.45 | 73.7 |
| % of duplicate reads (post-alignment) | 6.15 | 7.1 | 17.2 |
| % On Target Reads (post-alignment) | 67.4 | 59 | 70.1 |
| % Coverage >25X | 83.8 | 88.7 | 96.81 |
| % Coverage >50X | 48.3 | 61.58 | 73.1 |
| # of SNV | 191578 | 211623 | 230823 |
| # of Indels | 20730 | 31108 | 50139 |
